# Expression and prognostic value of PRDX family in colon adenocarcinoma by integrating comprehensive analysis and *in vitro* and *in vivo* validation

**DOI:** 10.3389/fonc.2023.1136738

**Published:** 2023-03-09

**Authors:** He Zhou, Lifa Li, Jia Chen, Songlin Hou, Tong Zhou, Yongfu Xiong

**Affiliations:** ^1^ The Second Department of Gastrointestinal Surgery, Affiliated Hospital of North Sichuan Medical College, Nanchong, China; ^2^ Institute of Hepatobiliary, Pancreatic and Intestinal Disease, North Sichuan Medical College, Nanchong, China; ^3^ Laboratory of Cancer Biology Department of Oncology, University of Oxford, Oxford, United Kingdom; ^4^ The Department of Hepatobiliary Surgery, Affiliated Hospital of North Sichuan Medical College, Nanchong, China

**Keywords:** peroxiredoxin family, bioinformatics analysis, biomarker, prognosis, colorectal cancer

## Abstract

**Background:**

The peroxiredoxin family, a crucial regulator of redox reactions, is strongly associated with various tumorigenesis. However, the role of peroxiredoxin4 (PRDX4) in colon adenocarcinoma (COAD) remains poorly understood.

**Methods:**

Multicenter databases, including GEPIA, HPA, UALCAN, cBioPortal, cancerSEA, STRING, CCLE, and LinkedOmics, comprehensively analyzed transcriptional expression, prognostic value, genetic alterations, signaling pathways, and associated genes of the PRDXs in COAD patients. Colony formation, transwell, flow cytometry, sphere formation, and xenograft assays were performed to validate further *in vitro* and *in vivo*.

**Results:**

Members of the PRDX family were differentially expressed in COAD, with each member showing varying degrees of genetic alterations. Intriguingly, only PRDX4 significantly correlated with COAD prognosis and stage. The single-cell sequencing suggested that PRDX4 is positively correlated with proliferation, apoptosis, and invasion, whereas negatively correlated with stemness. Moreover, PRDX4 involved in a series of critical biological processes, such as cell growth. Furthermore, *in vivo* and *in vitro* analyses indicated that knocking down PRDX4 inhibits the proliferation and invasion of HCT116 cells while promoting apoptosis and stemness.

**Conclusions:**

We identified PRDX4 expression as a novel potential prognostic marker in COAD.

## Introduction

Colorectal cancer (CRC) remains the third most common cancer and the second leading cause of cancer-related death worldwide, with approximately 1.9 million new cases and 900,000 deaths reported in 2020 (1). The incidence of CRC decreased in developed countries as early diagnosis, radiotherapy, and chemotherapy became more prevalent (2). In contrast, CRC incidence and mortality have rapidly increased in many low- and middle-income countries, such as Asia and South America (3). CRC still ranks among the top five tumor-related causes of death, and the incidence and mortality of CRC continue to increase annually in China, making it a significant public health concern (4, 5). Despite the various surgical-based treatments being applied to CRC, the 5-year survival rate of CRC patients in China is still not ideal (6). More than half of the patients were diagnosed in the middle and advanced stages and lost the chance of surgery.

Evidence suggests that CRC develops through a multi-step process, where abnormal cell proliferation is the initiator, and the invasion of advanced cancer cells is a leading cause of poor prognosis (7, 8). Currently, the diagnosis of CRC is mainly based on microscopic examination of tumor specimens, tumor node metastasis (TNM) staging is a crucial criterion for predicting the prognosis of CRC patients, and immunohistochemistry can be used to determine the colorectal origin of metastasis or visualize the spread of tumor cells in surrounding tissues. However, none of these means can diagnose CRC at an early stage. There is an urgent need to identify several highly sensitive and specific tumor markers and to intervene in the intracellular stage of tumorigenesis, which is critical for early prevention and improvement of the prognosis of CRC.

The reactive oxygen species (ROS) level in biological systems is critical for maintaining intracellular homeostasis (9). Emerging evidence has shown that excessive ROS can cause additional accumulation of DNA mutations leading to the initiation and progression of cancer (10). Several studies have demonstrated that antioxidants can maintain the balance between cell proliferation and apoptosis by reducing intracellular ROS levels to prevent tumorigenesis (11). Peroxiredoxins (PRDXs), a family of such antioxidants, catalyze the reduction of peroxides to maintain the balance of intracellular ROS levels (12). Currently, six PRDX isoforms have been identified in mammalian cells, namely PRDX1-PRDX6. They can be further divided into three subgroups: 2-cysteine (PRDX1-4), atypical 2-cysteine (PRDX5), and 1-cysteine (PRDX6), according to the mechanism of response to cysteine dependence.

There are few studies on the expression and prognostic value of the PRDX family in CRC. Thus, we performed a comprehensive bioinformatics analysis integrating multiple databases to systematically investigate the transcriptional expression profile, gene co-expression, cell line expression, survival prognosis, gene mutation analysis, and protein interaction network of the PRDX family in CRC. Intriguingly, the combined analysis revealed that only PRDX4 was significantly associated with the prognosis and various clinical characteristics of CRC patients. Consequently, we further validated the tumor-promoting properties of PRDX4 in CRC by *in vitro* and *in vivo* assays.

## Materials and methods

### Gene expression profiling interactive analysis

GEPIA (http://gepia.cancer-pku.cn/) is a newly developed interactive website that integrates TCGA and GTEX gene expression information, including extensive RNA raw sequencing expression data (13). Users can quickly find the expression of the target gene in the corresponding tumor by entering the gene symbol or Ensembl ID. In addition, GEPIA can also perform prognostic survival analysis, including two types of overall survival (OS) and disease-free survival (DFS). GEPIA can also analyze target genes and potential transcription factors by analyzing the correlation between two associated genes.

### The human protein atlas

The HPA database (https://www.proteinatlas.org/), dedicated to providing tissue and cellular distribution information for all 24,000 human proteins, is freely available for public inquiry (14). Users can query gene expression (RNA and protein expression) in various tissues by entering the target gene. According to the HPA database, we determined the expression of PRDXs in various solid tumors, immunohistochemical images of normal and cancer tissues from CRC patients, and the localization images of PRDXs in cells.

### The University of Alabama at Birmingham cancer data analysis portal

UALCAN (http://ualcan.path.uab.edu/) is a comprehensive and easily accessible web-based database built on TCGA (15). The database provides gene expression in different cancer tissues and corresponding normal tissues and allows comprehensive analysis of the association between target genes and multiple clinical characteristics. Our study utilized UALCAN to analyze the association between specific PRDXs and CRC clinic characteristics.

### Cancer single-cell state atlas

The CancerSEA database covers 41,900 single-cell functional annotation information of 25 types of tumors involving 14 cell functional states such as proliferation, invasion, and apoptosis (16). It enables users to investigate the relationship between the functional phenotypes of genes of interest and various tumors. For example, the CancerSEA was utilized to analyze the association between the PRDX family and CRC cell function.

### cBio cancer genomics portal

The cBioPortal database (https://www.cbioportal.org) combines 126 tumor genome studies, including the cancer cell line encyclopedia and TCGA (17). cBioPortal integrates genomic data such as somatic mutations, DNA copy-number alterations (CNAs), gene expression (mRNA, microRNA, protein), DNA methylation, and phosphorylated protein enrichment.

### Search tool for the retrieval of interaction gene

The String (https://www.string-db.org) is a database for searching the interactions between known proteins and predicted proteins. The database includes over 5,000 species, 24 million protein species, and over 20 million protein interaction links (18). In addition, we collected and integrated proteins and potential interactions related to PRDX family members by constructing a protein-protein interaction (PPI) network.

### LinkedOmics

The LinkedOmics database (http://www.linkedomics.org) includes multi-omics data and clinical data for 32 cancer types and data from 11,158 patients in the TCGA project (19). We can perform correlation analysis between target genes and other genes by inputting PRDX family genes, selecting cancer types and data sets, GO, KEGG, GSEA, and other analyses. Through this database, we can also obtain a series of visualization results, such as volcano plots of related genes and heat maps of positive and negative related genes.

### Cancer cell line encyclopedia

The CCLE is a database of cancer cell lines maintained by the Broad Institute of MIT, which currently has more than 1,000 cell lines (20). The PRDX4 expression data in CRC cells were obtained directly from the CCLE website (https://www.betastasis.com/). We evaluated the expression of PRDX4 in 57 CRC cell lines by CCLE.

### Cell culture and lentivirus transfection

HCT116 cells were purchased from the American type culture collection (ATCC) and cultured in RPMI-1640 medium (Hyclone, UT, USA) containing 10% fetal bovine serum (PAN-Biotech, Adenbach, Bavaria). The short hairpin RNA (shRNA) was synthesized by Sigma-Aldrich (NM_006406). The target sequence was as follows: PRDX4 5′-CCACACTCTTAGAGGTCTCTT-3′ (TRCN000064818, Sigma). In addition, the shRNA vector targeting GFP (SHC005, Sigma) was used as a knockdown control. We observed and photographed the cells using immunofluorescence microscopy, and the number of transfected cells more significant than 95% was considered successful. Moreover, RT-qPCR was applied to detect knockdown efficiency.

### RT-qPCR

We used RNA (Promega, Madison, USA) kit to extract the total RNA. The extracted RNA was then reverse-transcribed into cDNA using the PrimeScript™ cDNA synthesis kit (Takara, Japan). Finally, the results were calculated using the 2^-ΔΔCt^ method.

### Western blot

An extraction buffer containing protein lysis (Solarbio, Beijing, China) was used to obtain the total protein. Then the extracted protein was quantitatively analyzed using a BCA kit (Solarbio, Beijing, China). First, an equal amount of 30 μg proteins was added to each lane for electrophoresis and membrane transfer. Next, the protein bands were incubated with the primary antibody overnight at 4°C and the secondary antibody for one hour at room temperature. Finally, the western blot results were detected using the ECL chemical reagent method.

### Sphere formation assay

A key method for identifying cancer stem cells (CSCs) *in vitro* is their capacity to form spheres. Therefore, we used the sphere formation assay to assess the stemness of HCT116 cells in this work. First, cells were trypsinized to make a single-cell suspension, then seeded into six-well ultra-low cluster plates (4×10^3^ cells/well) and cultured in a serum-free medium (DMEM/F-12, Sigma, USA) containing 1% B27 (Gibco, USA), 0.02 μg/ml EGF (Peprotech, USA) and 0.02 μg/ml bFGF (Peprotech, USA) for 15 days. The sphere volume and number were observed under a microscope (Olympus, Japan) every four days, and a new medium was added.

### Colony formation, apoptosis, and transwell assay

The specific methods are consistent with our previous work (21). Briefly, for colony formation, PRDX4 knockdown and control cells were seeded into 6-well plates at a density of 300 cells/well and incubated in a cell incubator for two weeks. Then, the cells were fixed with 4% formaldehyde for 30 min and stained with crystal violet for 20 min. Colonies were counted under a microscope and images were collected. For apoptosis assay, the PRDX4 knockdown and control cells were disaggregated with trypsin and washed once with PBS. Then, 1 × 10^6^ cells were resuspended in PBS and stained using allophycocyanin and propidium iodide according to the manufacturer’s instructions. For transwell assay, the upper chamber of the 24-well plate was coated with diluted Matrigel and serum-free medium was added, and the lower chamber was filled with medium containing 20% FBS. The transfected cells (5 × 10^5^ cells/well) were seeded into the serum-free medium and incubated in a cell incubator for 48 h. Then, the cells were fixed and stained with 4% formaldehyde and 0.2% crystal violet, respectively. The invaded cells were photographed and counted under an inverted microscope.

### Xenograft assay and immunohistochemistry

The nude mice (BALB/c, four-week-old) were acquired from the Animal Center of North Sichuan Medical College. All experimental procedures were under the ARRIVE guidelines and approved by the Animal Ethics Committee of North Sichuan Medical College (No. SCXK2022-0018). All mice were randomly divided into two groups (sh-Control, n=5; sh-PRDX4, n=5). A total of 5×10^6^ cells were subcutaneously inoculated into each nude mouse. Tumor volume and weight were recorded every four days for four weeks after injection. After treatment, all mice were euthanized to isolate tumors, and tumor weights were measured. The immunohistochemistry (IHC) method is consistent with that described in our previous work (21).

### Statistical analysis

The web database generated all statistical analysis results, which were then calculated using the GraphPad Prism 9.0 software. Student’s t-test analyzed differences between the control and transfection groups. All experiments were repeated more than three times. *P* < 0.05 represents a statistical difference.

## Results

### Differential expression of PRDX family members in various tumors

**Figure 1:**
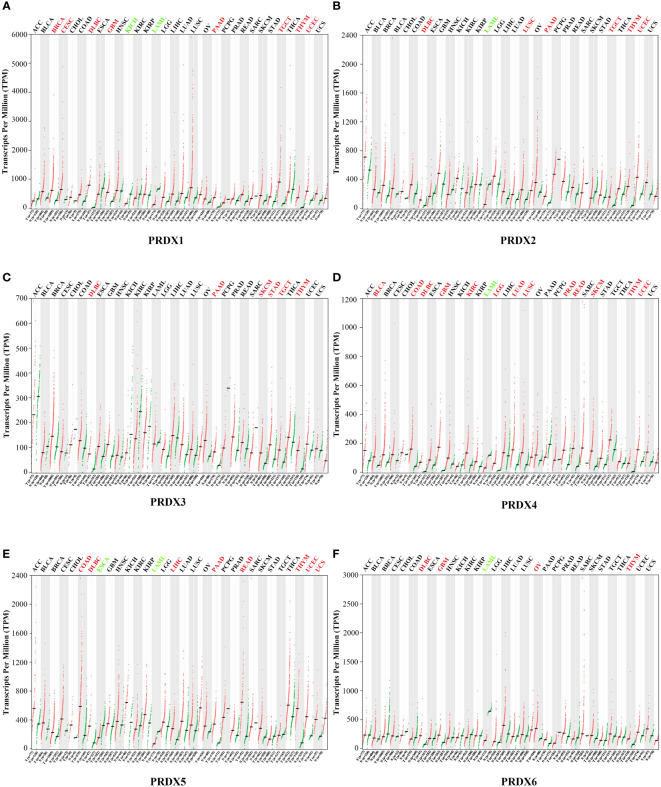
Expression of PRDX family members in various types of cancer (GEPIA). RNA-seq data for each PRDX family member in 31 cancer types are reported as TPM, generated by the TCGA. **(A-F)** Expression of PRDX1-6 in 31 cancers. The horizontal coordinate cancer name in red represents high expression, green represents low expression, and black represents no statistical significance. TPM, transcripts per million; TCGA, the cancer genome atlas.

**Figure 2:**
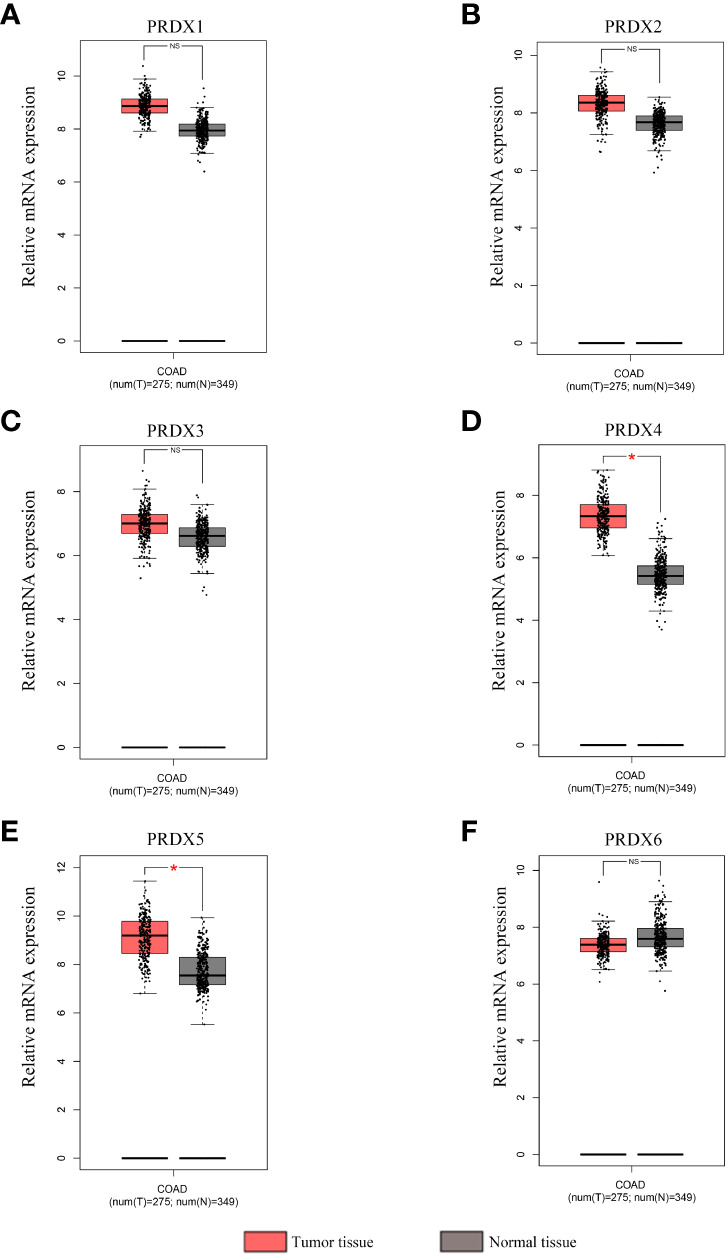
Expression analysis of PRDX family members in COAD and normal tissues (GEPIA). **(A-F)** The mRNA expression levels of each PRDX family member in COAD and corresponding normal tissues were compared in the box plot. *P < 0.05. NS, no significance; COAD, colon adenocarcinoma.

A total of 31 solid tumors were analyzed using the GEPIA database for the RNA expression of each member of the PRDX family (**Figure 1**). Specifically, PRDX1 was highly expressed in BRCA, CESC, DLBC, GBM, PAAD, TGCT, THYM, and UCEC, and lowly expressed in KICH and LAML (**Figure 1A**). PRDX2 was highly expressed in DLBC, LUSC, PAAD, TGCT, THYM and UCEC, while it was lowly expressed in LAML (**Figure 1B**). PRDX3 was highly expressed in DLBC, PAAD, SKCM, STAD, TGCT and THYM (**Figure 1C**). PRDX4 was highly expressed in BLCA, COAD, DLBC, GBM, KIRC, LGG, LUAD, LUSC, PRAD, READ, SKCM, THYM and UCEC, and was lowly expressed in LAML (**Figure 1D**). PRDX5 was highly expressed in COAD, DLBC, LIHC, PAAD, READ, THYM, UCEC and UCS, and low in ESCA and LAML (**Figure 1E**). PRDX6 was highly expressed in DLBC, GBM, OV and THYM and low in LAML (**Figure 1F**). Furthermore, we focused on the expression of PRDXs in COAD. Although PRDX1, PRDX2, and PRDX3 were highly expressed in COAD, their expression was not statistically significant (**Figures 2A-C**). PRDX6 was lowly expressed in COAD, but also not statistically significant (**Figure 2F**). Among the PRDX family, only PRDX4 and PRDX5 were highly expressed in COAD with statistical significance (**Figures 2D, E**).

**Figure 3:**
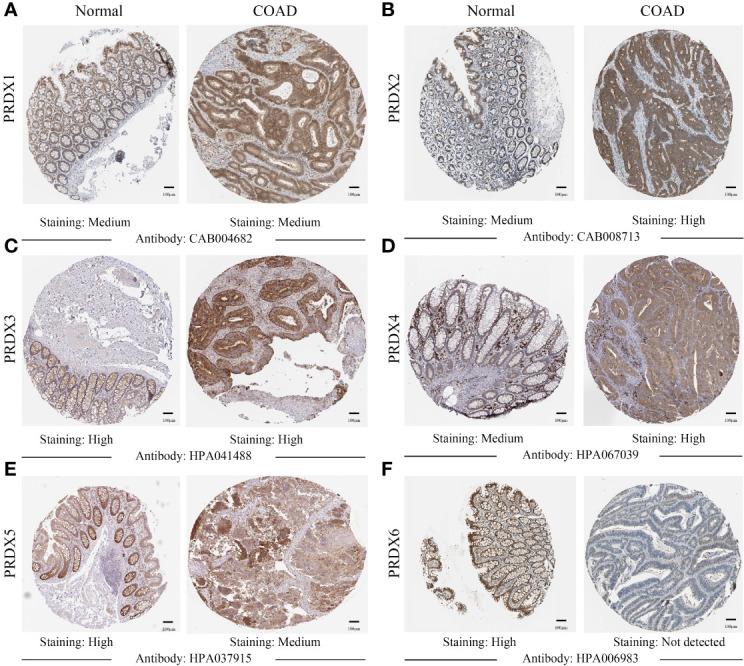
**(A-F)** IHC analysis of protein expression of each PRDX family member in COAD tissues and normal tissues (HPA). Brown areas in the image indicate positive expression, and blue areas indicate negative expression. IHC, immunohistochemical; COAD, colon adenocarcinoma. scale bar, 100 μm.

IHC results from the HPA database were used to analyze protein expression to confirm the PRDX family’s gene expression trends in CRC. Based on the degree of staining, the protein expression of PRDX2 and PRDX4 was significantly higher in colon cancer tissues than in normal tissues (**Figures 3B, D**). There is no significant difference in the protein expression of PRDX1 and PRDX3 in normal tissues and cancer tissues (**Figures 3A, C**). Conversely, the protein expression of PRDX5 and PRDX6 was lower in colon cancer tissues (**Figures 3E, F**).

**Figure 4:**
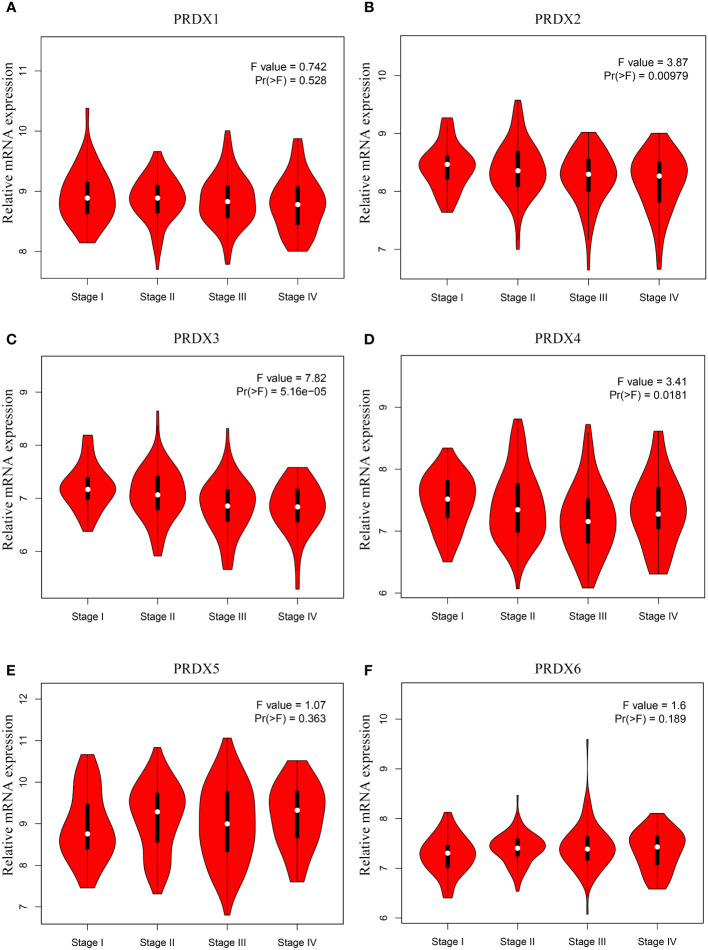
**(A-F)** Association between mRNA expression of PRDXs and tumor stage in COAD patients (GEPIA). The expression of PRDX2, PRDX3 and PRDX4 was significantly correlated with the pathological stage of COAD patients (*P* < 0.05). COAD, colon adenocarcinoma.

It is well known that tumor node metastasis (TNM) staging remains the crucial criterion for determining the prognosis of CRC patients. Therefore, we further evaluated the correlation between PRDX family members and CRC pathological stage. Interestingly, only the expression of PRDX2, PRDX3, and PRDX4 indicated significant variability (*P* < 0.05) (**Figures 4B-D**). In contrast, there was no correlation between PRDX1, PRDX5, and PRDX6 and the pathological stage of CRC (*P* > 0.05) (**Figures 4A, E, F**).

### Association between PRDX family expression and prognosis in CRC patients

**Figure 5:**
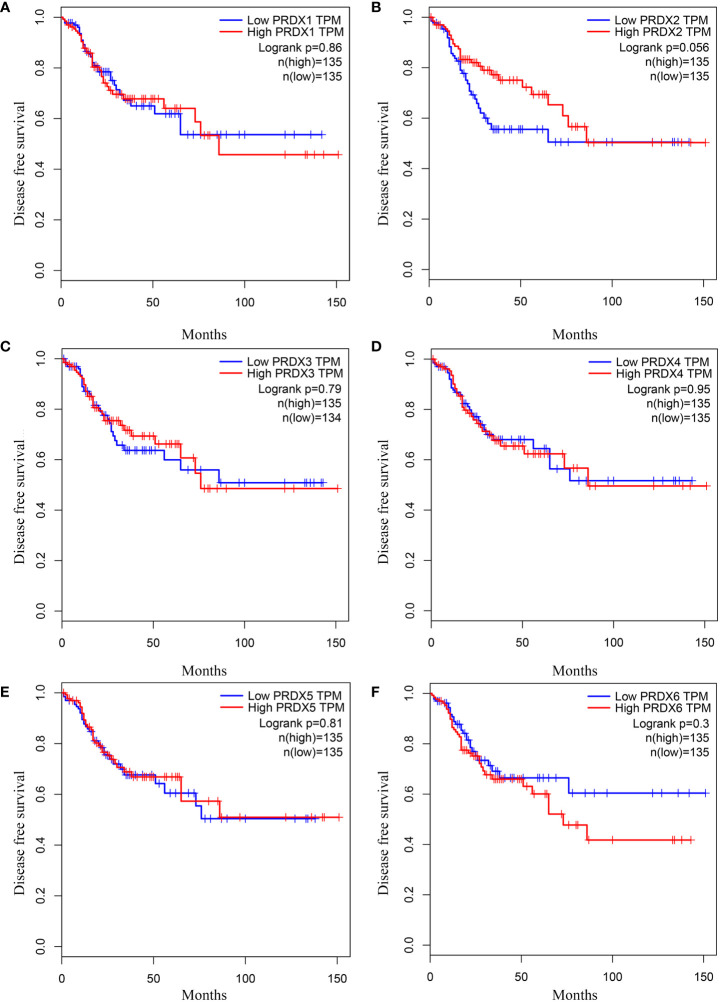
The DFS curves of PRDXs in COAD (GEPIA). **(A-F)** None of the PRDX family members were associated with DFS in COAD (*P* > 0.05). DFS, disease free survival; COAD, colon adenocarcinoma.

**Figure 6:**
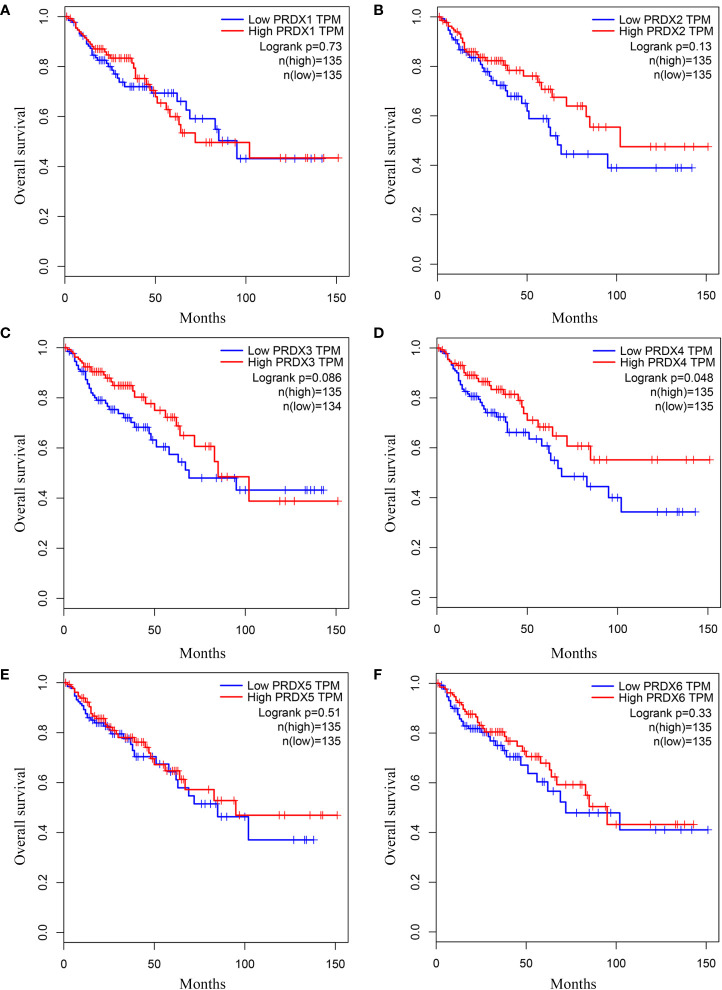
**(A-F)** The OS curves of PRDXs in COAD (GEPIA). **(D)** Only COAD patients with low expression of PRDX4 were significantly associated with shorter OS (*P* < 0.05). OS, overall survival; COAD, colon adenocarcinoma.

**Figure 7:**
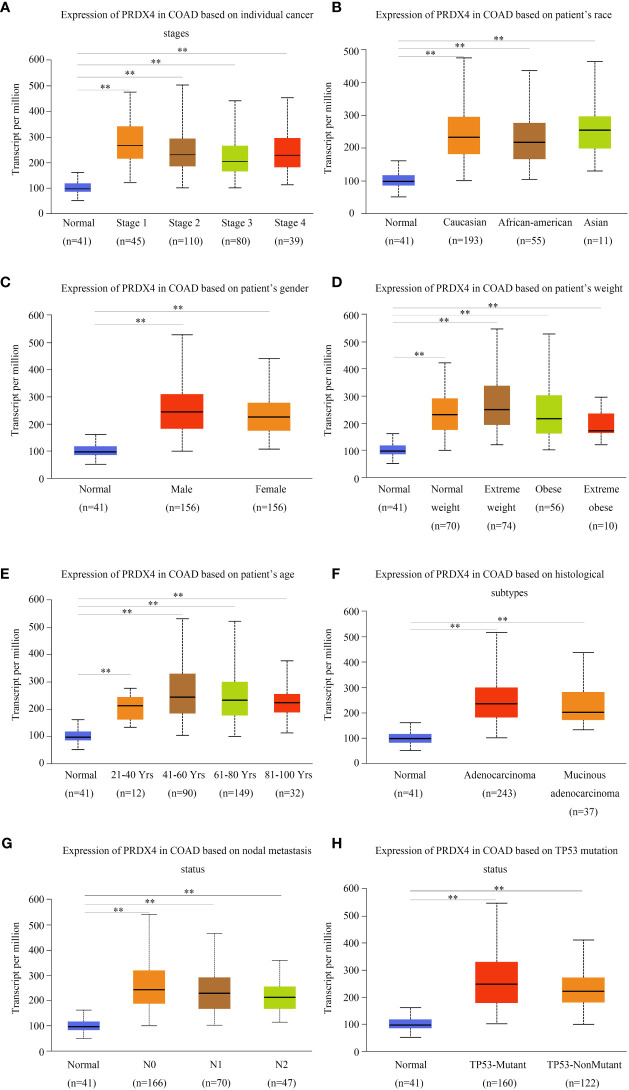
PRDX4 transcript levels in subgroups of COAD patients, stratified by individual cancer stages, race, gender, and other criteria (UALCAN). Compared with normal tissues, PRDX4 was highly expressed in all clinical subtypes shown in **(A-H)** and had a significant correlation with these clinical characteristics. **P < 0.01. COAD, colon adenocarcinoma.

We investigated the relationship between mRNA expression and OS and DFS in CRC patients using the GEPIA database. As shown in **Figure 5**, PRDX family members showed no significant difference with DFS. Surprisingly, only PRDX4 expression was statistically significant in terms of OS, and low PRDX4 expression was associated with a poor prognosis (**Figure 6D**). Therefore, we further analyzed the relationship between PRDX4 and the clinical characteristics of CRC patients in the UALCAN database. The results revealed that the expression of PRDX4 was significantly correlated with individual cancer stages, race, gender, weight, age, histological subtypes, metastasis status, and TP53 mutation status (*P* < 0.001) (**Figures 7A-H**). Given these findings, we speculated that PRDX4 might be a potential prognostic marker for CRC.

### Genetic alterations, protein expression and interaction analysis of PRDXs in CRC patients

**Figure 8:**
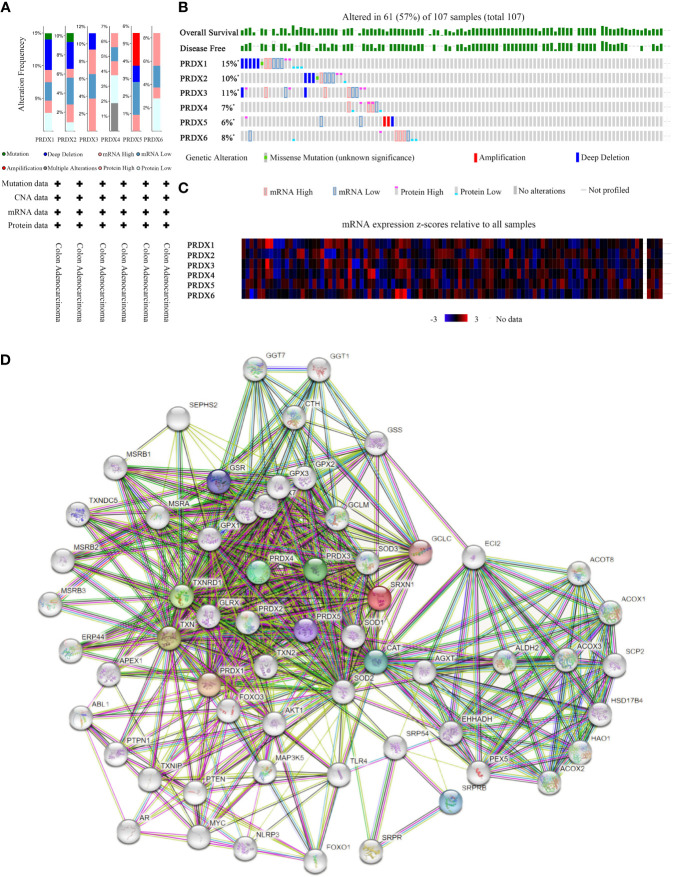
Mutation and expression analysis of PRDX gene in COAD (cBioPortal and STRING). **(A, B)** Summary of alterations in different expressed PRDXs in COAD. PRDXs were altered in 61 of the 107 COAD patient samples, accounting for 57%. **(C)** Heatmap showing mRNA expression z-scores relative to all samples (RNA Seq V2 RSEM UQ Log2). **(D)** Protein-protein interaction network of the PRDX family. COAD, colon adenocarcinoma.

PRDXs genetic alterations in CRC were analyzed using the cBioPortal database. The results revealed a minimum of four alteration types (PRDX3/5/6) and a maximum of six alteration types (PRDX1/2) (**Figure 8A**). Overall, PRDXs were altered in 61 of the 107 CRC patient samples (**Figure 8B**). Furthermore, PRDX1, PRDX2, PRDX3, PRDX4, PRDX5, and PRDX6 were altered in 15%, 11%, 10%, 7%, 6%, and 8% of CRC samples, respectively (**Figure 8B**). Moreover, the mRNA expression heatmap of PRDXs revealed that PRDX1 and PRDX3 had higher mutation rates (**Figure 8C**).

Furthermore, we performed PPI network analysis for each PRDX using the STRING to explore potential interactions between proteins. The results showed that there were 61 protein nodes related to the SFRP family in the constructed PPI network (**Figure 8D**).

### Functional states of the PRDX family in CRC cells

**Figure 9:**
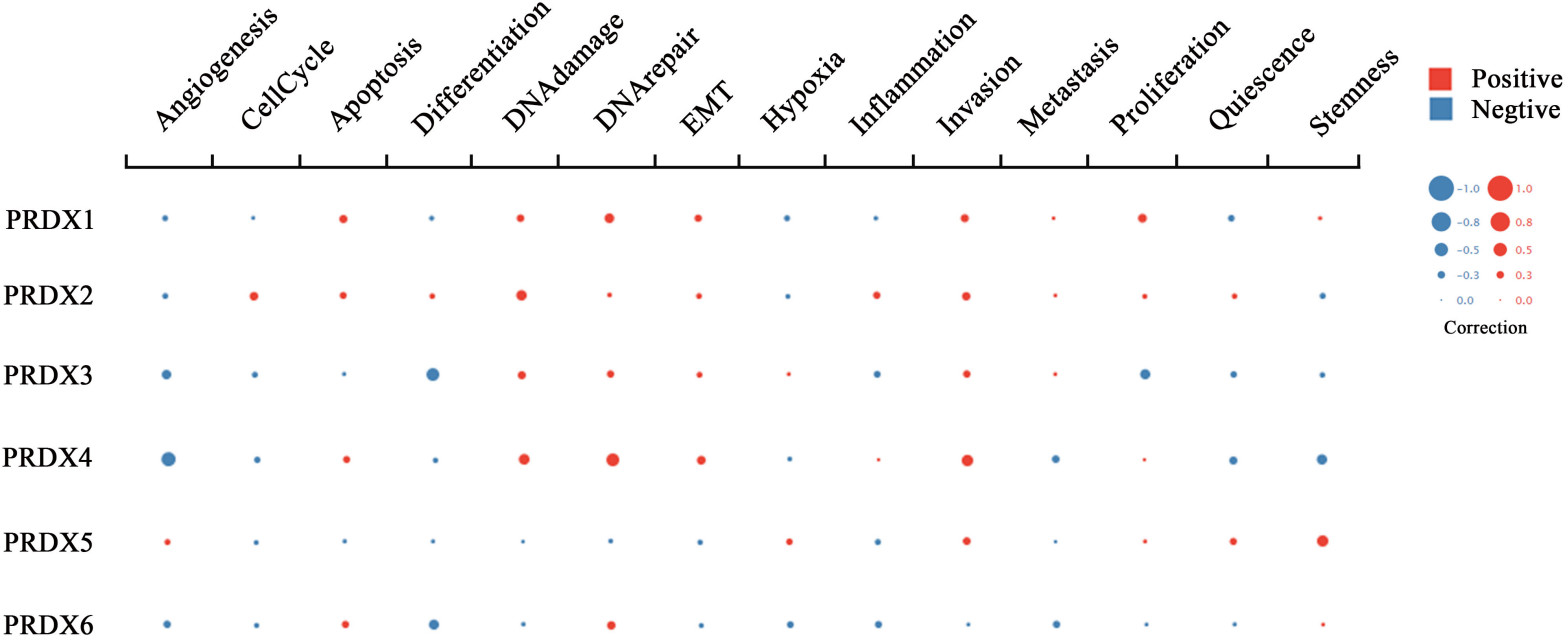
Functional state analysis of PRDX family members in CRC (cancerSEA). Functional relevance of each PRDX member in CRC cells. The size of the bubble indicates the strength of the correlation; red represents a positive correlation and blue represents a negative correlation. CRC, colorectal cancer.

RNA-seq technology’s emergence allows for exploring tumor cells’ functional heterogeneity. The association of each PRDX family member with 14 functional states in CRC cells is shown in **Figure 9**. As a potential clinical indicator of CRC progression, PRDX4’s role in cancer biology attracted our attention. According to cell function analysis, PRDX4 positively correlated with proliferation, apoptosis, and invasion of CRC cells but negatively correlated with stemness, which was closely associated with prognosis (**Figure 9**). Based on these results, it deserves to verify whether PRDX4 can have a similar effect on CRC cells by further *in vitro* analysis.

### KEGG pathway analysis and cellular localization of PRDX4-related differentially expressed genes in CRC

**Figure 10:**
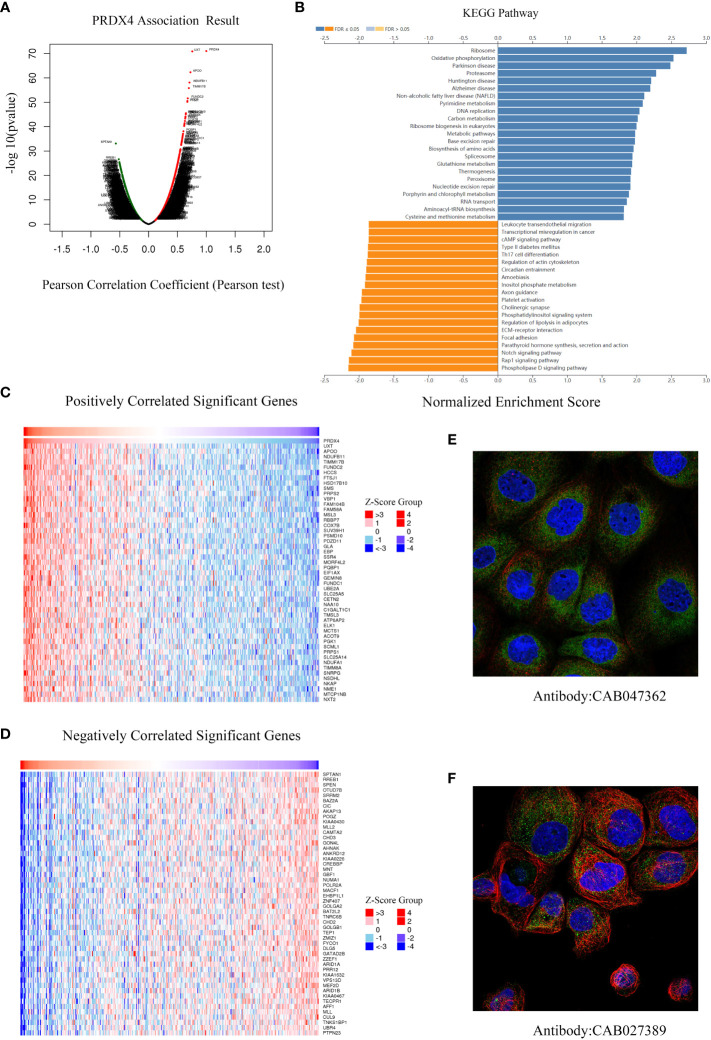
Differentially expressed genes associated with PRDX4 in CRC, KEGG pathway analysis and cellular localization (LinkedOmics). **(A)** Pearson test was used to analyze the correlation between PRDX4 and differentially expressed genes in CRC. **(B)** KEGG enrichment analysis showing functional pathways associated with PRDX4. **(C, D)** Heatmap analysis showing genes positively and negatively correlated with PRDX4 in CRC (Top 50). Red represents positively correlated genes, and green represents negatively correlated genes. **(E, F)** Subcellular localization of PRDX4. Green represents the target protein, blue represents the nucleus, and red represents the cellular microtubules. KEGG, kyoto encyclopedia of genes and genomes.

We further analyzed RNA-seq sequencing data of PRDX4 from 379 CRC patients using the LinkedOmics database. The results of the volcano plot displayed that 11628 genes (red dots) were positively correlated with PRDX4, while 8200 genes were negatively correlated (green dots) (**Figure 10A**). Moreover, **Figures 10C, D** show that 50 significant genes were positively and negatively correlated with PRDX4. Among them, the strongest positive correlation was found between PRDX4 and UXT (Pearson correlation = 0.76, *P* = 1.34e-71), whereas the strongest negative correlation was found with SPTAN1 (Pearson correlation = 0.57, *P* = 8.02e-34). Furthermore, KEGG pathway analysis showed that PRDX4 positively correlated with several major cellular functional processes, including DNA replication (**Figure 10B**). Moreover, through cellular localization analysis, we found that PRDX4 is mainly localized to the endoplasmic reticulum (**Figure 10E**). In addition, it is partially localized to the cytosol (**Figure 10F**).

### Differential expression of PRDX4 in CRC cell lines

**Figure 11:**
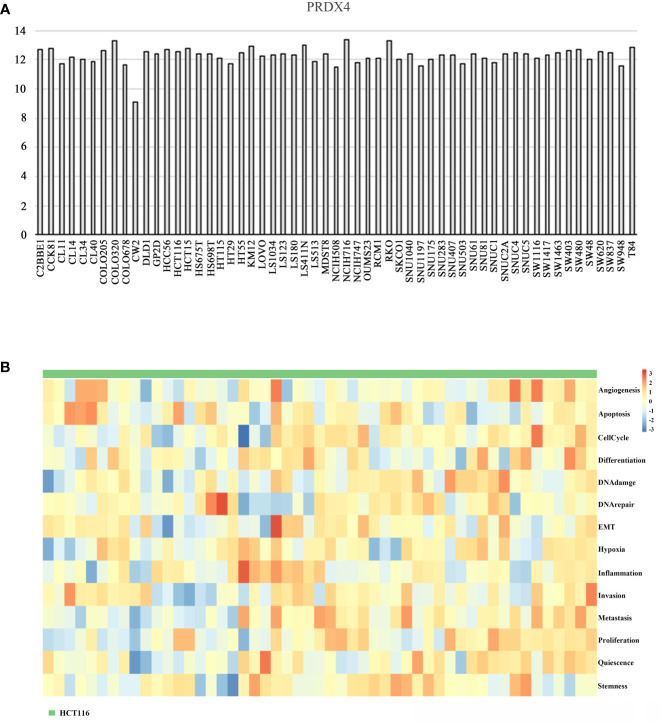
Differential expression of PRDX4 in different CRC cell lines (CCLE and cancerSEA). **(A)** The mRNA expression level of PRDX4 in various CRC cell lines, as determined by CCLE analysis. **(B)** The heatmap in this panel displays the activity of function states of cells in the selected dataset. Rows represent function states, columns represent hierarchically clustered cells. Column labels indicates the cell groups which cells belong to. CCLE, cancer cell line encyclopedia.

The CCLE database was applied to analyze the expression of PRDX4 in various CRC cell lines. The results suggested that among 57 CRC cell lines, the expression of PRDX4 in COLO320, HCT116, HCT15, KM12, LS411N, NCIH716, and RKO was significantly higher than in other cell lines (**Figure 11A**). Therefore, we selected one of the most common HCT116 cells for further *in vitro* and *in vivo* analysis. Furthermore, single-cell sequencing data (GSE81861) from the cancerSEA database revealed 14 functional cell states, such as apoptosis, proliferation, invasion, and stemness, in HCT116 cells without prior treatment (**Figure 11B**).

### Knockdown of PRDX4 inhibits CRC cell proliferation and invasion, but promotes apoptosis and stemness

**Figure 12:**
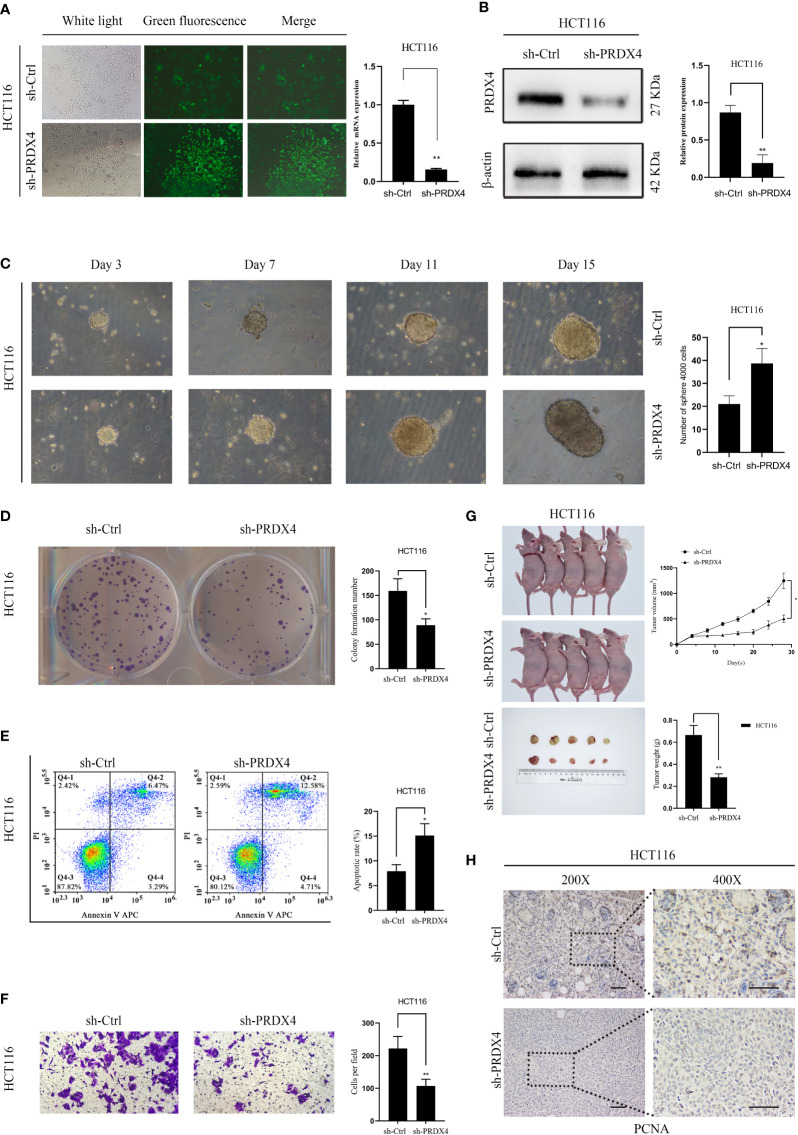
Knockdown of PRDX4 inhibits CRC cell proliferation and invasion and promotes CRC cell stemness and apoptosis. **(A)** The transfection efficiency was observed by fluorescence microscopy and detected by RT-qPCR. **(B)** Western blot was used to validate the expression of PRDX4 protein in HCT116 cells transfected with shRNA. **(C)** Sphere formation assay of HCT116 cells showing that knockdown of PRDX4 promoted cell stemness. **(D)** Colony formation assay of HCT116 cells showing that knockdown of PRDX4 inhibited cell proliferation. **(E)** Flow cytometry results showing that knockdown of PRDX4 promoted apoptosis in HCT116 cells. **(F)** Transwell assay of HCT116 cells showing that knockdown of PRDX4 inhibited cell invasion. **(G)** Knockdown of PRDX4 inhibited tumor growth of HCT116 cells *in vivo*. **(H)** Images of IHC staining of PCNA in xenograft tumors. scale bar, 50 μm. *P < 0.05, **P < 0.01. IHC, immunohistochemical; PCNA, proliferating cell nuclear antigen.

Further analyses of PRDX4 cellular functions were performed both *in vitro* and *in vivo* to validate the cancer SEA database findings. Firstly, HCT116 cells with stable knockdown expression PRDX4 were established by lentiviral transfection (**Figure 12A**). RT-qPCR and western blot assays were then performed to validate the knockdown efficiency of the lentivirus. Both mRNA and protein levels were significantly reduced by targeting shRNA to PRDX4 (**Figures 12A, B**).

Numerous studies have found a strong link between the stemness of cancer cells and the prognosis of cancer patients (22). Consequently, a sphere formation assay was utilized to determine the effect of PRDX4 expression level on the stemness of cancer cells. The results showed that knocking down PRDX4 dramatically promoted the sphere-forming capability of cancer cells (**Figure 12C**). As shown in **Figures 12D–F**, knocking out PRDX4 inhibited HCT116 cell proliferation and invasion while promoting apoptosis. Notably, the experimental results of PRDX4 apoptosis were not consistent with the bioinformatics analysis in **Figure 9**. Furthermore, the effect of PRDX4 on cancer cell proliferation *in vivo* was assessed by injecting sh-Ctrl or sh-PRDX4 cells into nude mice. The results suggested that the tumor volumes were significantly reduced in the sh-PRDX4 group compared to the control group (**Figure 12G**). Proliferating cell nuclear antigen (PCNA) was used to indicate tumor proliferation. IHC staining revealed that the expression of PCNA in the sh-PRDX4 group was significantly lower than in the control group (**Figure 12H**). Taken together, these results demonstrate the involvement of PRDX4 in regulating CRC cell progression.

## Discussion

Recent studies have noted that ROS, a crucial regulator of the intracellular environment, is strongly associated with tumorigenesis (23). In addition, ROS is involved in the growth process of tumor cells and can promote metastasis and thus affect patient prognosis (24). This has led to the suggestion that ROS modulators may be helpful in the primary prevention of cancer.

Increasing research has demonstrated that aberrantly expressed PRDXs are involved in a series of tumor cell processes, including cell proliferation, apoptosis, and invasion (25, 26). For instance, several studies have indicated that PRDX1 can function as a pro-oncogene in lung cancer (27) and soft tissue sarcoma (28). *In vivo*, PRDX1 knockout mice developed several malignancies, including lymphomas and sarcomas (29). In addition, PRDX1, PRDX2, and PRDX6 have also been reported to be overexpressed in oral squamous cell carcinoma (30–32). Moreover, the tumor-promoting role of PRDX4 has been demonstrated in lung cancer, leukemia, and glioblastoma (33–35). As for PRDX5, it shows a dual function in tumors. Elamin et al. found that PRDX5 was the only member of the PRDX family markedly downregulated in breast cancer (36). In contrast, Gérard et al. reported the tumor-promoting role of highly expressed PRDX5 in thyroid cancer (37). Surprisingly little is known about the PRDX family in CRC, particularly PRDX4. Therefore, we conducted this study to address this issue.

PRDX was first introduced in 1994, and subsequent studies found that PRDX4 is located on human chromosome 10p22.13 (38, 39). Numerous studies have demonstrated the potential of PRDX4 as a biomarker for many diseases, such as diabetes and stroke (40). However, the role and prognostic value of PRDX4 in CRC remain unknown, which drew our attention. To our knowledge, this is the first study to combine multicenter databases to analyze the expression and prognostic value of the entire PRDX family in CRC. The data in this study included cell lines and clinical samples and comprehensively revealed the expression and prognostic value of the PRDX family in CRC. First, we found that PRDXs are differentially expressed in colon adenocarcinoma (COAD) at the mRNA level. Consistently, PRDXs showed the same expression trend at the protein level in COAD. Then we explored the relationship between PRDXs and tumor stage and found that only PRDX2, PRDX3, and PRDX4 were significantly correlated with COAD stage. Finally, GEPIA further investigated the prognostic value of PRDXs in COAD. Nevertheless, only PRDX4 showed a significant correlation in OS. Moreover, we also found that PRDX4 was associated with multiple clinical characteristics, including age, sex, pathological subtype, lymph node metastasis, etc., suggesting that PRDX4 may be a potential prognostic marker for COAD.

Recently, several studies have identified that mutations cause approximately 40% of CRC cases in tumor suppressors or oncogenes (41). Further genetic analyses revealed that differentially expressed PRDXs undergo frequent genetic alterations in COAD. These data could partially explain the causes of colorectal carcinogenesis. By building a protein interaction network, we found that PRDXs are mostly associated with peroxides and cell proliferation proteins. We further focused on the functional states of PRDXs in CRC. The results suggested that PRDX4 was positively associated with apoptosis, proliferation, and invasion and negatively associated with stemness. We discovered that PRDX4 is primarily involved in the biological process of cell proliferation using KEGG enrichment analysis, which is consistent with previous research. Moreover, our *in vitro* and *in vivo* experiments further validated PRDX4’s role in COAD. In HCT116 cells, knocking down the expression of PRDX4 inhibited proliferation and invasion and promoted apoptosis. These findings are consistent with the studies reported by Yi et al. (42). Extensive studies have demonstrated the involvement of CSCs in developing tumors, and their formation is one of the leading causes of cancer recurrence (43). Intriguingly, the downregulation of PRDX4 expression promotes stemness in HCT116 cells. This finding may explain the poor prognosis caused by the low expression of PRDX4. Overall, our study systematically reveals the primary role of PRDXs, especially PRDX4, in the carcinogenesis of COAD and provides a promising strategy for treating COAD.

Our study has some undeniable limitations. All analytical data in our study were obtained from online databases, and additional clinical samples must be collected for subsequent validation. Furthermore, our *in vitro* and *in vivo* assays used only one type of COAD cell, and our findings need to be validated in more CRC cells.

## Conclusion

Our current research mainly has the following innovations: (1) We used multiple databases for the first time to systematically examine the transcriptome, mutation, mRNA, and protein of the PRDX family in CRC. (2) The differential expression of PRDX4 was finally determined by screening to be closely related to the prognosis of COAD patients. (3) Our *in vitro* and *in vivo* analyses revealed that aberrant expression of PRDX4 has significant effects on the process of COAD, including proliferation, apoptosis, invasion, and stemness. Based on our comprehensive analysis and validation, PRDX4 may represent a new prognostic factor for COAD.

## Data availability statement

The datasets presented in this study can be found in online repositories. The names of the repository/repositories and accession number(s) can be found in the article/**Supplementary Material**.

## Ethics statement

The animal study was approved by the Animal Ethics Committee of North Sichuan Medical College (license number: SCXK2022-0018).

## Author contributions

HZ and YX designed the research, LL performed the experiments, SH, JC, and TZ analyzed the data, HZ wrote the article, and all authors revised and approved the manuscript. All authors contributed to the article and approved the submitted version.
